# A systematic review of antibacterial activity of polyphenolic extract from date palm (*Phoenix dactylifera* L.) kernel

**DOI:** 10.3389/fphar.2022.1043548

**Published:** 2023-01-10

**Authors:** Raman K. Bhaskaracharya, Archana Bhaskaracharya, Constantinos Stathopoulos

**Affiliations:** ^1^ Department of Food Science, College of Agriculture and Veterinary Medicine, United Arab Emirates University, Al-Ain, United Arab Emirates; ^2^ Nepean Blue Mountains Local Health District/ University of Sydney, Sydney, NSW, Australia; ^3^ Faculty of Agrobiology, Food and Natural Resources, CZU Prague, Prague, Czech Republic

**Keywords:** date kernel extract, polyphenols, antibacterial, minimum inhibitory concentration, minimum bactericidal concentration, ethnopharmacology, medicinal plants, antimicrobial

## Abstract

**Background:** Emergence of antibiotic-resistant bacteria makes exploration of natural antibacterial products imperative. Like other fruit processing industry by-products, date kernels, a waste from date processing industry is rich in its extractable polyphenols. The rich polyphenolic content suggests that date kernel extracts (DKE) can be a cost-effective source of antimicrobial agents, however, their antibacterial activity is poorly understood. Hence, a systematic review of available literature to establish DKE’s antibacterial activity is warranted.

**Methods:** A systematic PRISMA approach was employed, and relevant studies were identified using defined keywords from Google Scholar, Scopus, PubMed, and Web of Science databases. The search results were screened based on predefined eligibility criteria and data extraction, organization, pooling, and descriptive statistical analyses of original research records conducted.

**Results:** A total of 888 published records were retrieved from databases. Preliminary screening by applying specific eligibility criteria reduced records to 96 which after full text screening further decreased to 14 records. *Escherichia coli* and *Staphylococcus* a*ureus* were the most studied organisms. Results indicate moderate to highly active effect shown by the less polar solvent based DKE’s against Gram-positive and by the aqueous based DKE’s against Gram-negative bacteria. The review confirms antibacterial activity of DKE against both Gram-positive and -negative bacteria. Heterogeneity in reported polyphenolic content and antibacterial activity are due to differences in cultivars, extraction methods, test methods, model organisms, *etc.* Use of standardized protocols for isolation, characterization, testing of DKE’s active polyphenols to elucidate its antibacterial activity is recommended to establish the clinical efficacy of natural antibacterial compounds from DKE.

**Conclusion:** This review outlines the current knowledge regarding antibacterial activity of polyphenolic DKE, identifying gaps in information and provides key recommendations for future research directions.

## 1 Introduction

The emergence of antibiotic-resistant bacteria has refocussed scientific exploration for natural antibacterial products. With the potent antibacterial activity of polyphenols well established, the by-products of fruit processing industry such as pomace and kernels are excellent source for polyphenols and could be used as a cost-effective, alternative antibacterial agents with acceptable potency (Krivokapić et al., 2021). Their mechanism of action is by inhibiting cell wall formation, altering the cytoplasmic membrane permeability, changing the ability of bacterial cell to attach to substrate or damaging nucleic acid synthesis (Abdullah et al., 2017). Date palm kernels with their rich polyphenolic content, are excellent candidates to mine antibacterial agents.

Date palm (*Phoenix dactylifera* L.) is a major fruit tree in most of Arabian Peninsula which provides date palm fruit, that have been used over the centuries for various purposes. Date fruits are common to most markets and are consumed all over the world. Kernels from date fruits, also referred to as seeds or pits, account for 13%–15% of the date fruit weight (Selim et al., 2022). Date kernel, a by-product/waste, from date processing industry are an excellent source of fibre, fat, proteins, ash and are rich in phytocompounds namely, phenolics, anthocyanins, carotenoids, tocopherols, tocotrienols, etc (Bouaziz et al., 2013). Studies have shown that date kernels contain higher amounts of phenolic content and antioxidants in comparison to their fruit (Al-Farsi and Lee, 2008). Such phytocompounds can be easily mined, however are currently underutilised and wasted. Valorisation of date kernels obtained from waste stream of date processing industries, to use as a functional food ingredient, has attracted increasing scientific attention (Mrabet et al., 2020).

The ethnomedical usage of *Phoenix dactylifera* L. (date palm) is well known and it has been part of human diet since antiquity (Sadeghi & Kuhestani, 2014; Nazri et al., 2016; Selmani et al., 2017). Dates palm fruits along with its products such as dry dates, date butter, date jam, date syrup, date bars, date candy and date juice concentrate are consumed globally. Date kernels have been incorporated into bakery products and chocolate (Platat et al., 2015) while roasted date kernels are proposed as an excellent decaffeinated alternative to coffee (Venkatachalam and Sengottian, 2016). A nutraceutical drink containing polyphenols from date kernel powder has also been developed as a therapy against chronic diseases (Ahmed et al., 2017).

The antibacterial activity of DKE although studied lacks from commercial application due to conflicting efficacy claims (Hussain et al., 2019; Metoui et al., 2019; Radfar et al., 2019). A comprehensive and systematic literature search is warranted to determine effectiveness of DKE as an antibacterial agent. The methodologies applied for antibacterial testing, microorganisms employed for investigation, representation of results, extraction methods applied, phytocompounds identified in the studies *etc.*, needs to be reviewed methodically (Al-Daihan and Bhat, 2012; Bhat and Khalaf, 2012). The current study, therefore, systematically reviews, all available literature published until March 2022 to ratify the antibacterial potential of polyphenolic extract from date kernels based on current knowledge.

## 2 Research questions

Polyphenolic compounds extracted from plants, besides their established antioxidant activity, exhibit significant antibacterial activity (Bouarab-Chibane et al., 2019).

This systematic review evaluates if-

1. Available literature conclusively can determine that polyphenols from DKE are effective against spoilage and pathogenic bacteria.
2. Claims of antibacterial activity of DKE polyphenols have been validated by employing standard protocols.
3. Stratification of DKE’s antibacterial activity can be based on extraction-method and/or associated to date palm cultivars.

## 3 Methods and materials

### 3.1 Search strategy

The literature search from inception to March 2022 was undertaken from three academic databases (Scopus, PubMed, and Web of Science) and secondary sources (Google Scholar and Google search engine) following the Preferred Reporting Items for Systematic Reviews and Meta-Analyses [PRISMA] guidelines (Page et al., 2021). Keywords employed for literature search included polyphenol, antibacterial, antimicrobial, total phenolic content, total phenols content, phenolic profile, phenolic composition, date seed, date palm seed, date kernel, date palm kernel, date pit, date palm pit, and *Phoenix dactylifera*. The search included various combinations of Boolean operators (OR, AND, NOT) to combine the search keywords and find relevant records. List of cited references from the selected records were manually reviewed to include additional records. Duplicate records were removed and non- English records or deemed to be non-topic related records were excluded. Pre-set eligibility criteria were applied and only original research (reviews were eliminated) was included. Data collected from included studies were organized, pooled, and analysed using descriptive statistics.

### 3.2 Eligibility and inclusion/exclusion criteria

Specific inclusion criteria comprised of the following:

1) primary research studies published in scientific journals that are in English language,
2) studies on the antibacterial activity of DKE, and
3) presence of polyphenolic compounds has been assessed and reported.

Studies were excluded that met any of the predetermined exclusion criteria comprising of the following:

1) review articles that did not include original research,
2) studies which did not use DKE (*Phoenix dactylifera* L.) in microbiological tests,
3) studies where the antibacterial activity of DKE was assessed in conjunction with other antimicrobials to evaluate synergism, and
4) studies dealing with nanoparticles sourced from date kernels.

### 3.3 Data extraction and organization

A predesigned data extraction form was employed by one of the researchers (RKB), with the aim of answering the guiding research questions of this systematic review and verified by the second researcher (AB). In the case of disagreements, consensus was reached by discussion and based on factual evidence. The PRISMA statement (Page et al., 2021) was referred for data organization and evaluation.

### 3.4 Assessment of risk of bias

The risk of bias (ROB) was assessed by all authors using a standard approach and in accordance with guidance criteria for evaluating *in-vitro* studies with some adjustment (Lynch et al., 2016). Fourteen parameters were identified as guidance criteria for quality assessment of final list of records for focussed evaluation.

Broadly, these quality assessments include the standard criteria of the definition of the issue, the identification of purpose and hypothesis, the study design, the quality of the methodology for data collection, data analysis and manuscript drafting. If the report described the quality assessment criteria, the article received a “Y’’ (yes) on that specific parameter, if it was missing information, the article received an “N’’ (no). The scoring from all three reviewers were collated, averaged and the ROB calculated. Records that reported 0–five items were considered as having high ROB (score +++), those reporting six to nine items as having medium ROB (score ++), and those reporting 10–14 items as having low ROB (score +).

### 3.5 Statistical analysis

Data collation was conducted using Microsoft Excel. GraphPad Prism 9.3.1 was used for data analysis and graphical representation. Descriptive statistics was employed, and collated data was expressed as median ± range and mean at the 95% confidence interval (CI).

## 4 Results and Discussion

### 4.1 Study selection

**FIGURE 1:**
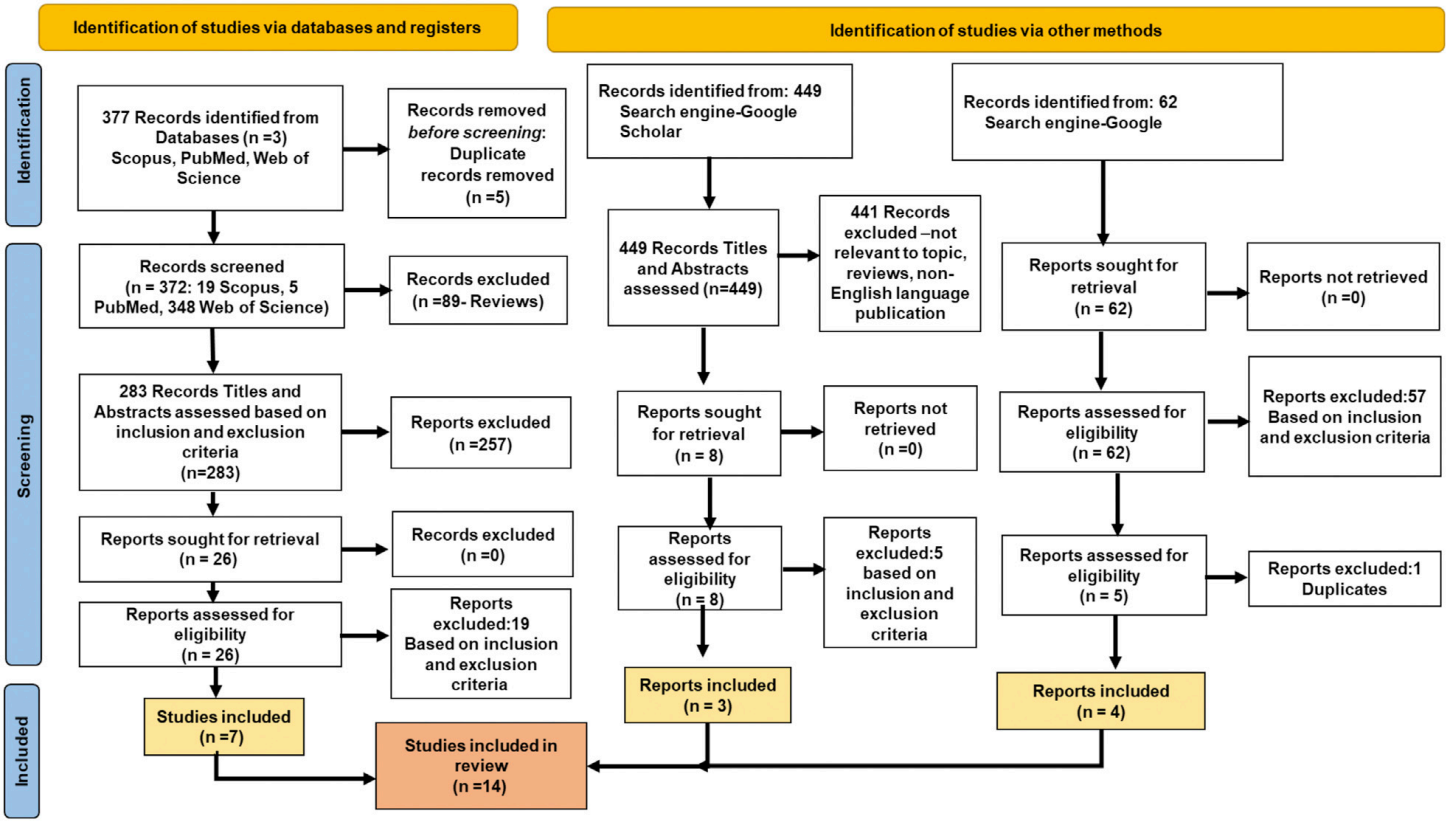
Search Strategy- PRISMA flow diagram.

The PRISMA flow diagram (Figure 1) describes stepwise approach of selecting research records for this review. A combined total of 888 records were identified from primary databases- Scopus, PubMed and Web of Science and secondary sources namely, Google scholar and Google search engine. Of the 377 records identified from databases (Scopus, PubMed, Web of Science), five duplicate records, 89 review articles and 257 non-topic related records were excluded. Out of the 26 records retrieved, 19 records did not meet the pre-set eligibility criteria. Hence 7 records were included for detailed analysis, data collation and statistics. Search from Google Scholar yielded 449 records from which 441 records were eliminated as non-topic specific records, review articles and were in non-English language. The 8 remaining records were assessed and of these only 3 records met the pre-set eligibility criteria. Furthermore, Google Search Engine yielded 62 records from which 57 records were eliminated for meeting exclusion criteria and/or not meeting inclusion criteria and 1 record for duplication, leaving 4 records for inclusion in this study. Thus, of a total of 888 records, only 14 records which met our pre-set eligibility criteria were evaluated for risk of bias (ROB).

### 4.2 Risk of bias assessment

**FIGURE 2:**
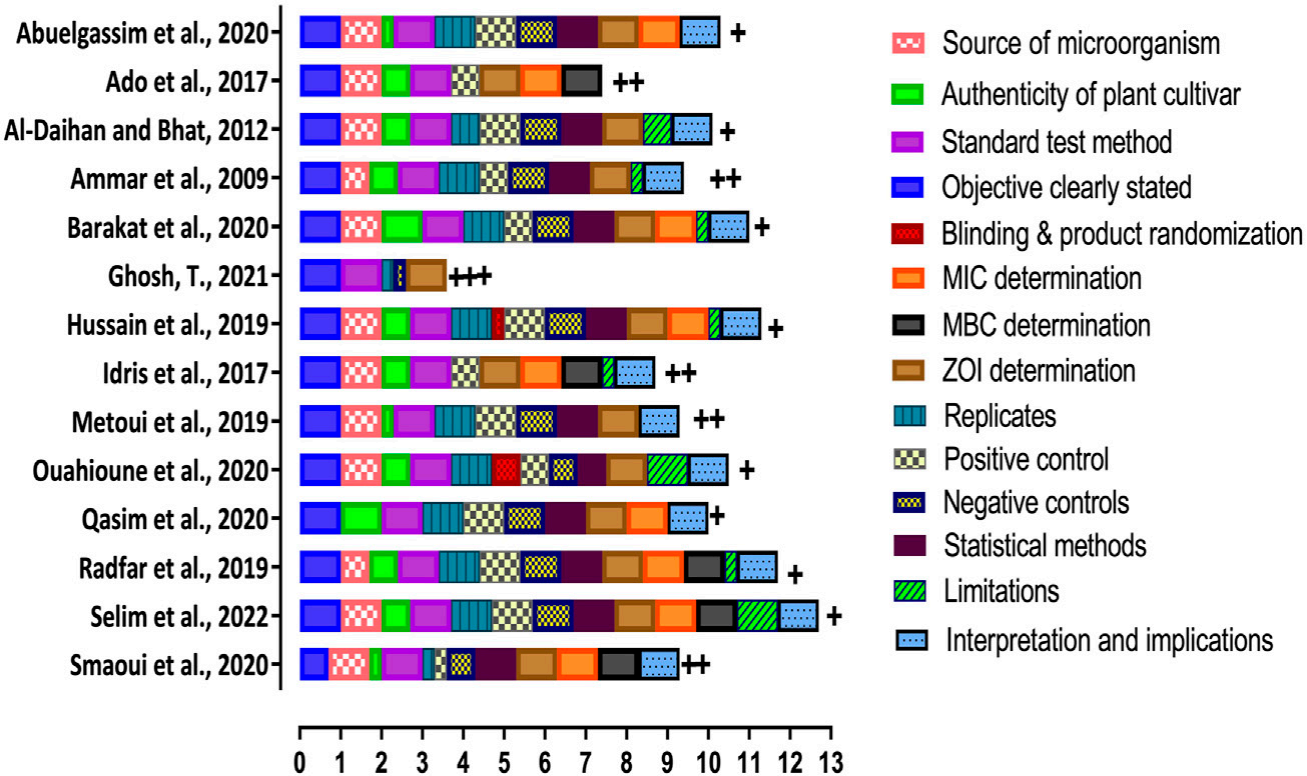
Risk of bias (ROB) assessment. Articles were assessed based on whether the records have reported. the source of microorganism, authenticity of plant cultivar, standard test method usage, clear statement of objective of the study, blinding and product randomization, MIC determined, MBC determined, ZOI determination, replicate analyses was performed, positive and negative control usage, statistical methods used for data analyses, identified limitations of their study and providing the interpretation and implications of the observed outcomes. Each of the quality assessment criteria is identified as a different coloured box. The size of the box is determined by the mean of the assigned scores given by the three authors for that parameter. After rounding the mean total scores to nearest whole number, the ROB of the selected studies was calculated as follows: Reporting 0–5 items - high ROB (score +++); Reporting six to nine items - medium ROB (score ++); Reporting 10–14 items - low ROB (score +).

Prior to data collation and statistical analysis, the 14 records finalised for inclusion in this systematic review were evaluated for quality and classified into high, medium, and low ROB. The assessment was conducted by the three authors at outcome level (Figure 2). Among the 14 studies, 1 received high ROB (score +++), 5 received medium ROB (score ++), and 8 received low ROB (score +). In case the mean score for a parameter fell between the classes, then the rounded mean value to the nearest whole number was used. One study, Ghosh (2021), scored 3.5 (high ROB) and was excluded from further detailed analysis. The remaining 13 records scored between 7 and 11 (out of 14) and were used for data synthesis and formulation of conclusions.

### 4.3 Study characteristics and variations

This review aimed at systematically collating available information regarding the antibacterial activity of polyphenolic DKE and focused on evaluating if the claims of antibacterial activity of DKE polyphenols have been validated by employing established standard protocols.

The presence of polyphenols was set as a prerequisite inclusion criterion to determine the antibacterial potential of DKE. Among the final 13 records included in this review, the presence of polyphenols was characterised by TPC, TFC, qualitative phytochemical analysis, and chromatographic profiling while the antibacterial activity was mainly measured as MIC, MBC and ZOI (10 studies used agar well diffusion while three studies used disc diffusion method).

**FIGURE 3:**
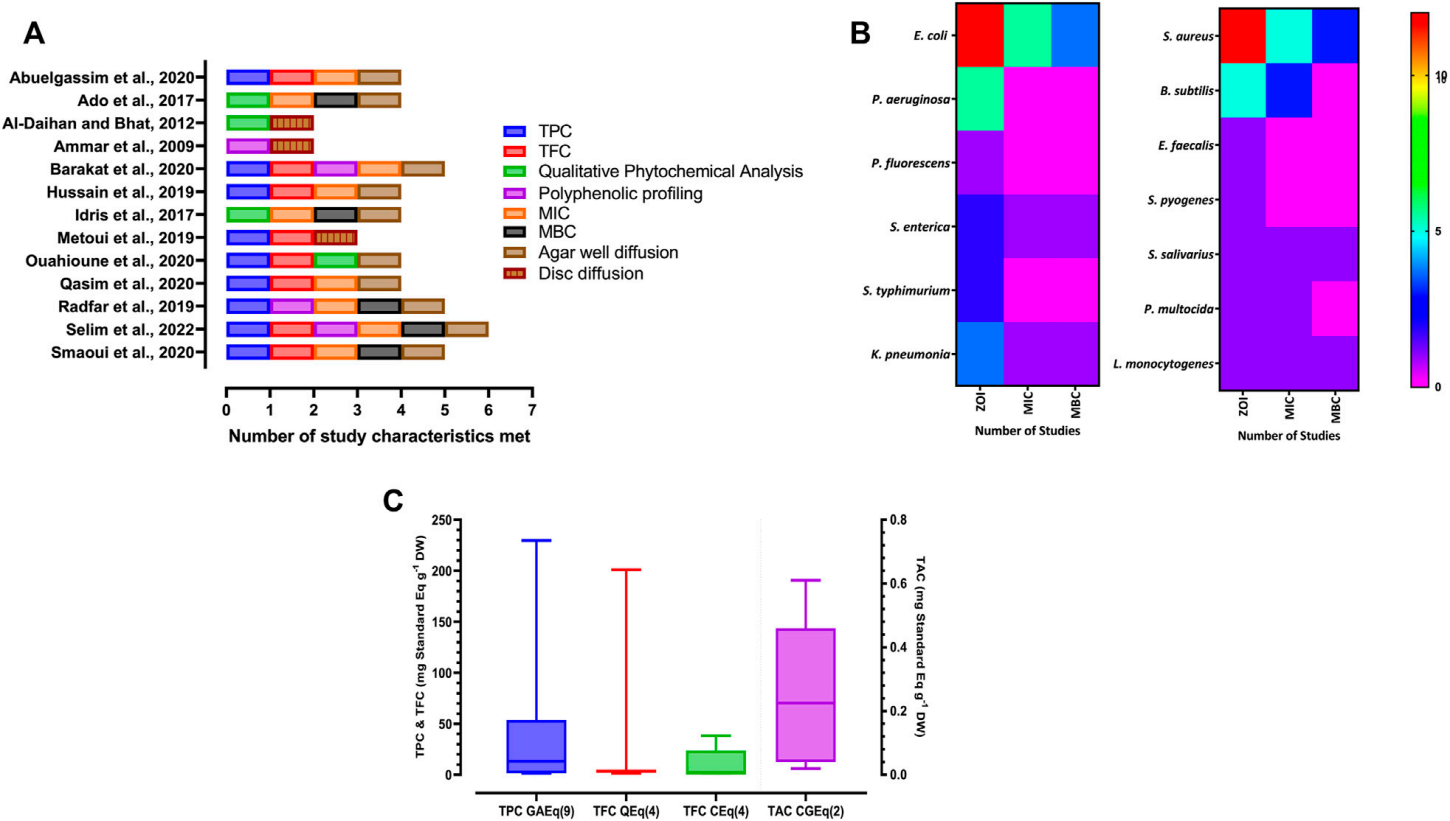
**(A)** Study characteristics and analysis variations. **(B)** Heatmap of antibacterial assay characteristics reported for gram-negative and gram-positive bacteria in included studies. **(C)** Boxplot of pooled median ± range of total phenolic content (TPC), total flavanoid content (TFC) and total anthocyanin content (TAC) values reported in included studies (values in parentheses indicate number of studies).

The review of the 13 records showed heterogeneity in the analysed and reported characteristics. Potential antibacterial activity was attributed in 4 records, to the presence or absence of phytochemical content in DKE (Table 1). Remaining 9 records assessed TPC, 8 of which also assessed TFC (Figure 3A). In some records, extraction, and chromatographic isolation of polyphenolic compounds from DKE was reported. Among the included records, analytical variations in quantitative or qualitative determinations of polyphenols were noted.

**TABLE 1:** Phytochemical screening results of included studies.

	Ouahioune et al. (2020)	Ado et al. (2017)	Idris et al. (2017)	Al-Daihan and Bhat, (2012)
Cultivar	Degla-Baida	Not Described	Not Described	Mosaifah
Type of Extract	Aqueous	Ethanolic	Ethanolic	Crude Powder
Saponnins	Present	Present	Present	Not Detected
Alkaloids	Present	Present	Present	Present
Terpenoids	Present	Not Tested	Not Tested	Not Tested
Glycosides	Not Tested	Present	Present	Not Tested
Steroids	Not Tested	Not Detected	Not Detected	Present
Tannins	Not Tested	Not Detected	Not Detected	Not Detected
Flavonoids	Not Tested	Not Tested	Not Tested	Not Detected
Anthraquinones	Not Tested	Not Tested	Not Tested	Not Detected
Catechin	Not Tested	Not Tested	Not Tested	Not Detected
Phenolic groups	Not Tested	Not Tested	Not Tested	Not Detected

Diffusion assays were employed to assess the antibacterial potential of polyphenolic DKE in all 13 included studies (Figure 3A). MIC and MBC were reported in 9 and 5 of the 13 included studies, respectively (Figure 3A). Additional assays namely time-kill studies and biofilm inhibition assay were conducted in one study each by Selim et al. (2022) and Qasim et al. (2020) respectively to assess the antibacterial activity of DKE. While most studies reported results of antibacterial activity assays performed in triplicate, two studies did not report the number of replicates assayed.

It is recommended that common pathogenic strains consisting of at least a Gram-positive and a Gram-negative bacterium must be used for screening plant extract’s antibacterial activity (Cos et al., 2006). Among the 13 included records, most common Gram-negative test organism employed were *Escherichia coli* (12 records) and *Pseudomonas aeruginosa* (5 records) while those for Gram-positive organisms were *Staphylococcus aureus* (11 records) and *Bacillus subtilis* (4 records). Other organisms against which DKE’s antibacterial activity has been tested include *Klebsiella pneumonia* (2 records), *Enterococcus faecalis* (2 records), *Salmonella typhimurium* (2 records), *Salmonella enterica* (1 record), *Streptococcus salivarius* (1 record), *Streptococcus pyogenes* (1 record), *Staphylococcus epidermidis* (1 record), *Pseudomonas fluorescens* (1 record), *Serratia marcescens* (1 record), *Proteus vulgaris* (1 record), *Listeria monocytogenes* (1 record), and *Pasteurella multocida* (1 record). With the emergence of antimicrobial resistance as a global threat to human health, a “priority status” has been designated by WHO to ESKAPE pathogens for developing new antimicrobials or complementing alternative therapies (Oliveira et al., 2020). Therefore, assessment of DKE’s antibacterial activity against these organisms is of a special interest in the present-day context.

Standardization is recommended to minimize inter and intra laboratory bias due to multiple factors that can affect the outcome of *in-vitro* susceptibility assays (Church and Naugler, 2019). The results of antibacterial activity of DKE were reported mostly as ZOI followed by MIC and the least reported as MBC for any test organism (Figure 3B). Standardized isolates were used in only 8 of the 13 included studies, while the remaining 6 studies either sourced clinical isolates from local culture banks or did not report the source of the microorganisms (Table 2). Furthermore, most of the included studies, sourced bacterial strains from recognised microbial culture collections meeting the standard recommendations (Cos et al., 2006), however, variations among the bacterial strains employed was noted (Table 2). This complicates collation of published results as an organism’s sensitivity to antibacterial agent varies between different isolates of even the same microbial species (Eloff, 2019). Furthermore, lower susceptibilities of clinical isolates of bacteria have been reported to plant extracts as compared to those with known resistance phenotypes (Ullah et al., 2016; Masota et al., 2021).

**TABLE 2:** Overview of selected records reporting antibacterial activity of polyphenols sourced from date kernel extracts.

References	Type of extract*	Bacteria
Abuelgassim et al. (2020)	Met	*S. aureus* (clinical isolate); *B. subtilis* NCTC 10400; *E. faecalis* ATCC 29212; *E. coli* NCTC 10418; *S. typhimurium* ATCC 13311 and *P. aeruginosa*
Ado et al. (2017)	*E*th	*E. coli* (clinical isolate)
Al-Daihan and Bhat, (2012)	Aq, Met, Ace	*S. aureus*, *S. pyogenes*, *E. coli* and *P. aeruginosa*
Ammar et al. (2009)	Met	*S. aureus*; *B. subtilis*; *E. coli* and *P. fluorescens*
Barakat et al. (2020)	Met, *E*th, Ace	*E. coli* O157-H7 ATCC 51659 and *P. aeruginosa* NRRL B-272; *S. aureus* ATCC 13565 and *B. subtilis* BTN7A
Hussain et al. (2019)	Ace, *E*th, EtAce, Hex, Met: Chl	*S. aureus* ATCC 29213 and *E. coli* ATCC 25922
Idris et al. (2017)	*E*th	*E. coli* and *S. aureus* (clinical isolate)
Metoui et al. (2019)	Aq, Ace, Met	*E. coli* ATCC 35218, *S. typhimurium* ATCC 1408, *E. faecalis* ATCC 29212, *S. aureus* ATCC 25923 and *S. epidermis* CIP 106510
Ouahioune et al. (2020)	Aq	*S. aureus* ATCC 25923, *E. coli* ATCC 25922, *K. pneumoniae* ATCC 4352, and *P. aeruginosa* ATCC 27853
Qasim et al. (2020)	*E*th	*B. subtilis* and *P. multocida*
Radfar et al. (2019)	Hex & *E*th	*S. aureus* ATCC 29213 and *E. coli* O157:H7 ATCC 35218
Smaoui et al. (2020)	*E*th, Ace, Ace: *E*th: Aq mix (4 ratios)	*L. monocytogenes* ATCC 19115, *S. aureus* ATCC 6538, *S. enterica* ATCC 14028 and *E. coli* ATCC 25922
Selim et al. (2022)	Met	*S. salivarius* ATCC 25975, *S. aureus* ATCC 19701, *S. marcescens* ATCC 99006, *E. coli* ATCC 29998, *K. pneumoniae* ATCC 13883, *P. vulgaris* ATCC 8427 and *P. aeruginosa* ATCC 10145

Abbreviations used: *Met- Methanol; Eth- Ethanol; Aq- Aqueous; Ace- Acetone; EtAce-Ethyl Acetate; Hex-Hexane; Chl- Chloroform.

Among all the included studies, false positives due to aspecific cytotoxicity was ruled out by testing against an array of test organisms. However, plant extracts are not always safe and plant-derived products can be a potential source of deleterious side effects (Njeru and Muema, 2021). Therefore, to ensure that the antibacterial activity of DKE is within the acceptable toxicity and selectivity index limits, it is imperative to evaluate its cytotoxicity in the dosage employed for antibacterial activity testing (Afagnigni et al., 2020). The evidence regarding antibacterial activity was not fortified by cytotoxicity data in any of the included studies.

The concentration/dose of extract tested for antibacterial activity varied among the included studies. Eloff (2019), postulates that if the dose is high enough, all plants have antimicrobial activity. Therefore, it is imperative that the concentration/dose information be furnished when detailing antibacterial activity. However, several studies included in this systematic review failed to report the concentration used for determining ZOI and the DKE concentration (Table 3) also varied among the included studies. Crude polyphenolic extracts were employed for antibacterial testing in all but one study. Selim et al. (2022) employed purified gallic acid for antibacterial activity testing. While the authors established purity by detecting the FTIR, ^1^H and ^13^C NMR spectra, the percentage purity of the extracted gallic acid was not reported.

**TABLE 3:** Key parameters of antibacterial assays in included studies.

Study reference	Bacterial media	Diffusion assay—test dose	Control	Assay method (AWD/DD)	Incubation (°C/h)	MIC methodology
Abuelgassim et al. (2020)	MHA	ND	Ampicillin-sulbactam, ceftazidime. Dose- ND	AWD	ND/ND	ND; References quoted
Ado et al. (2017)	NA	10 μg- 1 mg/ml	Ciprofloxacin (30 μg/ml)	AWD	37 °C/24 h	Tube dilution method- Tubes without turbidity recorded as MIC.
Al-Daihan and Bhat, (2012)	NA	100 μL/disc	Kanamycin (30 μg/disc)	DD	37 °C/24 h	ND
Ammar et al. (2009)	Lauri-Bertani Agar	100 μg	Ampicillin Trihydrate- dose ND	DD	37 °C/24–48 h	ND
Barakat et al. (2020)	MHA	50 μg GAE	ZOI- ND; MIC calculated	AWD	37 °C/18 h	Agar dilution diffusion method, minimum dose of DKE- expressed as MIC
Hussain et al. (2019)	ND (CLSI)	10 mg/ml	Amikacin (30 μg)	AWD	37 °C/24 h	Performed but ND
Idris et al. (2017)	NA	10 μg- 1 mg/ml	Ciprofloxacin (30 μg/ml)	AWD	37 °C/24 h	Tube dilution - Tubes without turbidity were recorded as MIC.
Metoui et al. (2019)	Mueller-Hinton agar	7.5 mg/disc	Ciprofloxacin & Lamidaz (100 mg/disc). Oxacillin (500 mg/disc)	DD	37 °C/24 h	ND
Ouahioune et al. (2020)	MHA	75 μl of 100 mg/ml	Gentamycin Dose- ND	AWD	4 °C/4 h; 37 °C/18–24 h	ND
Qasim et al. (2020)	ND-NCCLS	100 µl of 10 mg/ml	Ampicillin Dose- ND	AWD	ND/ND	ND
Radfar et al. (2019)	MHA	10 μl of 200 mg/ml	Ampicillin (10 μg)	DD	37 °C/24 h	Broth microdilution- turbidity
Smaoui et al. (2020)	MHA	50 μl of 20 mg/ml	Not Used	AWD	37 °C/24 h	Broth microdilution- colour change due to indicator
Selim et al. (2022)	NA	5 mg/disc of Gallic acid from Ajwa DKE	Amoxicillin, Gentamycin & Streptomycin (30 g/disc)	AWD	37 °C/24 h	Broth microdilution and plating dilutions as cfu growth

Abbreviations: AWD- agar well diffusion; CLSI- clinical and laboratory standards institute protocol; DD- disc diffusion; MHA- Mueller-Hinton Agar; NA- nutrient agar; NCCLS- national committee for clinical laboratory standards; ND- not determined.

Factors such as differences in the isolates employed, the use of clinical isolates, test methods used, DKE concentration tested *etc.*, may all limit the interlaboratory reproducibility of data. To objectively evaluate DKE’s antibacterial activity, a thorough reporting of key aspects of the extraction protocol, drug concentration used, polyphenolic and antibacterial activity evaluation parameters, is recommended along with the use of standard methods from bodies like EUCAST and CLSI. The heterogeneity of data and gaps in reporting parameters precluded a comprehensive meta-analysis, therefore, a descriptive statistical analysis was instead performed in this review.

### 4.4 Results of syntheses

The three research questions focussed in this review are, to evaluate if available literature can conclusively determine that polyphenols from DKE are effective against key spoilage and pathogenic organisms. Secondly, to assess if the antibacterial activity claims of DKE polyphenols have been validated by employing standard protocols and thirdly, stratification of the antibacterial activity of DKE based on extraction-methods and/or associated date palm cultivars.

#### 4.4.1 Analysis of polyphenolic content in DKE

Phytocompound subclasses such as phenolic acids, phenols, flavonoids, flavones, flavonols, quinones, tannins and coumarins are important for developing potential antimicrobial therapeutics (Farhadi et al., 2019; Hemeg et al., 2020; Ble-González et al., 2022). In 4 of the 13 included studies, qualitative phytochemical screening of DKE identified differences in presence of saponins, alkaloids, terpenoids, glycosides, steroids, tannins, flavonoids, anthraquinones, catechin and phenolic groups (Table 1). Ouahioune et al. (2020) reported presence of alkaloids, saponins, and terpenoids in aqueous Degla-Baida DKE while, Ado et al. (2017) and Idris et al. (2017) reported presence of alkaloids, saponins and cardiac glycosides in ethanolic DKE sourced (date palm cultivar is unknown). Al-Daihan and Bhat (2012) reported presence of alkaloids and steroids in the crude powder of Mosaifah cultivar but did not detect saponins (Table 1). As previously reported in literature, these variations could be attributed to the differences in the cultivars and extraction methods (El-Mergawi et al., 2016; Al-Alawi et al., 2017).

TPC in 9 of the 13 selected studies reported as gallic acid equivalents (GAE) were recalculated as mg GAE g^−1^ DW for comparison. The TPC of DKE ranged from 0.05 (Hussain et al., 2019) for Abu Maan and Mabroom cultivars to 229.7 mg GAE g^−1^ DW (Ouahioune et al., 2020) for Degla-Baida cultivar with the median TPC (Figure 3C) estimated as 13.24 mg GAE g^−1^ DW (n = 46; Mean = 31.52 mg GAE g^−1^ DW and Lower- Upper 95% CI of mean = 18.72–44.33 mg GAE g^−1^ DW). In line with previously reported literature, a huge variation in TPC values between cultivars was observed (Supplementary Figure S1) however, these findings must be interpreted cautiously as only one to two records were available for each cultivar (Bouhlali et al., 2020).

The TFC was reported in four studies as Quercetin Equivalent (QE) while four studies employed Catechin Equivalent (CE) as reference standard. TFC values were segregated according to the reference standard employed for data collation. The median TFC (Figure 3C) was calculated as 3.45 mg QE g^−1^ DW (*n* = 14; Mean = 17.39 mg QE g^−1^ DW and Lower- Upper 95% CI of mean = −13.16–47.93 mg QE g^−1^ DW) and 2.9 mg CE g^−1^ DW (n = 28; Mean = 11.63 mg CE g^−1^ DW and Lower- Upper 95% CI of mean = 6.21–17.05 mg CE g^−1^ DW).

The TAC was evaluated in only 2 of the included studies which employed pH differential method to report values as Cyanidin-3-glucoside (CGE) equivalent. The calculated median TAC (Figure 3C) was 0.23 mg CGE g^−1^ DW (*n* = 22; Mean = 0.24 mg CGE g^−1^ DW and Lower- Upper 95% CI of mean = 0.15–0.33 mg CGE g^−1^ DW).

Data heterogeneity made the stratification and pooling of data especially challenging. The TPC and TFC of Degla Baida cultivar’s aqueous extract was reported to be as high as 229.67 mg GAE g^−1^ DW and 201.12 mg QE g^−1^ DW respectively (Ouahioune et al., 2020). In contrast, the reported values of TPC and TFC of Sukkari (20.14 mg GAE g^−1^ DW and 0.84 mg QE g^−1^ DW respectively) and Khalas (20.6 mg GAE g^−1^ DW and 0.95 mg QE g^−1^ DW respectively) cultivars was almost ten times lower (Abuelgassim et al., 2020) shown in Table 4 and Supplementary Figure S1.

**TABLE 4:** Total polyphenolic, total flavonoid and total anthocyanin contents reported in the studies included in this systematic review.

References	Extract Type^a^	Cultivar	TPC^b^	TFC^c^	TAC^d^
Abuelgassim et al. (2020)	Ace-Aq + Bu-Aq for TPC/TFC analysis	Sukkari	20.14	0.84 A	
		Khalas	20.60	0.95 A	
Barakat et al. (2020)	Met	Abreme	8.40	2.07 B	
	*E*th		11.80	3.13 B	
	Ace		9.30	2.7 B	
	Aq		1.80	0.81 B	
Metoui et al. (2019)	50% Ace	Lemsi	51.30	14.3 B	0.25
		Amari	81.40	32.2 B	0.32
		Hammouri	65.00	19.8 B	0.46
		Korkobi	94.10	38.3 B	0.48
		Matata	71.30	23.9 B	0.53
		Halwaya	78.50	25.2 B	0.38
		Rochdi	87.20	32.3 B	0.46
		Deglet Nour	71.40	23.1 B	0.61
		Baht	52.20	15.4 B	0.2
		Bouhattam	58.20	16.3 B	0.26
		Eguiwa	94.80	36.1 B	0.41
		Khadhouri	95.30	36.8 B	0.46
Hussain et al. (2019)	50% *E*th, 50% Ace, EtAce, Met: Chl (1:1)	Khalas	0.07	0.04 B	
		Lulu	0.06	0.02 B	
		Fard	0.06	0.02 B	
		Ajwa	0.08	0.05 B	
		Abu Maan	0.05	0.04 B	
		Mabroom	0.05	0.04 B	
Ouahioune et al. (2020)	Aq	Degla-Baida	229.67	201.12 A	
Qasim et al. (2020)	20% *E*th	Khalas	0.48–1.21	0.21–0.74 B	
		Ajwa	0.90–1.40	0.24–0.83 B	
Radfar et al. (2019)	Hex +80% *E*th	Kabkab	33.77		
		Zahedi	33.1		
		Rabbi	24.23		
		Mazafati	14.83		
Smaoui et al. (2020)	Ace	Deglet Nour	9.92	3.92 A	0.04
	*E*th		8.25	2.97 A	0.04
	Aq		5.81	2.17 A	0.02
	50% *E*th		14.33	2.11 A	0.03
	50% Ace		13.83	5.07 A	0.03
	1:1 Ace: *E*th		10.29	2.90 A	0.06
	1: 1: 1 Ace: *E*th: Aq		15.97	4.55 A	0.06
	4.7: 1: 1 Ace: *E*th: Aq		12.65	5.69 A	0.05
	1: 4.7: 1 Ace: *E*th: Aq		11.52	3.94 A	0.04
	1: 1: 4.7 Ace: *E*th: Aq		8.14	1.84 A	0.03
Selim et al. (2022)	80% Met	Ajwa	24.84	5.324 A	

Abbreviations used.

^a^ Ace- acetone; EtAce-Ethyl Acetate; Eth- ethanol; Aq- Aqueous; Hex-Hexane; Met- Methanol; Bu- butanone.

^b^ TPC- total polyphenolic content as Gallic Acid Equivalent (mg GAE g^−1^ DW).

^c^ TFC- Total flavonoid content as (A) Quercetin Equivalent (mg QE g^−1^ DW) or as (B) Catechin Equivalent (mg CE g^−1^.DW).

^d^ TAC-Total Anthocyanin content as Cyanidin 3-Glucoside Equivalent (mg CGE g^−1^ DW).

Apart from the cultivar associated differences, interlaboratory variations of polyphenolic content may also be attributed to differences in extraction protocols. Najjar et al. (2022) reported variations in TPC of Khalas cultivar (range from 0.39 to 0.82 mg GAE g^−1^ DW) when extracted in water under different sample: solvent ratio levels. Such heterogenicity was also identified in this review wherein the anthocyanin content of 50% aqueous acetone extract from Deglet Nour was reported to be 20 times higher by Metoui et al. (2019) than the TAC content reported by Smaoui et al. (2020) using similar extraction solvents.

Therefore, several sources of variations including interlaboratory results, differences in extraction methods, solvents employed, and cultivars assessed, seem to be contributing to the heterogeneity of reported polyphenolic content in DKE. Similar findings were also reported by AlFaris et al. (2021) in their systematic review of TPC in date palm fruit. Apart from the cultivar type and extraction procedure, numerous other factors such as geographic location, climate, irrigation, sunlight, harvest time, post-harvest treatments, maturity, and experimental conditions are reported to affect the date palm phenolic composition (AlFaris et al., 2021) which may contribute to the variations observed in our study.

Chromatographic extraction and isolation of polyphenols was undertaken in 4 of the included studies. Radfar et al. (2019) reported varying content of gallic acid, vanillic acid, 3,4 dihydroxy benzoic acid, 2,5 dihydroxy benzoic acid, cinnamic acid, caffeic acid, chlorogenic acid in the four cultivars (Rabbi, Zahedi, Kabkab and Mazafati) employed in their study. The presence of gallic acid and caffeic acid was also detected in Abreme cultivar along with protocatechuic acid, p-hydroxybenzoic acid, catechin, chlorogenic acid, syringic acid, rutin and kaempferol (Barakat et al., 2020). Selim et al. (2022) isolated hydroxybenzoic acids-syringic acid, gallic acid, pyrogallol, quinol; hydroxycinnamic acids-ferulic acid, p-coumaric acid, vanillic acid, caffeic acid; flavones-apigenin, luteolin and flavonols-myricetin, quercetin from Ajwa’s extract. In another study, Ammar et al. (2009) identified flavonoid constituents isoquercetrin, luteolin 7-O--d-neohesperopyranoside 3-O-methylether, luteolin 7-O--d-neohesperopyranoside, acacetin 7-O--d-neohesperopyranoside, apigenin 7-O-d-apiofuranoside, apigenin seven- O-d-apiofuranosyl-(1 2)-O--d-glucopyranoside and genistein 8-C--d-glucopyranoside in aqueous methanolic extract of Balah Meghal cultivar. Among the antibacterial activity studies, Selim et al. (2022) investigated for Gallic acid isolated from Ajwa DKE while, Radfar et al. (2019), Barakat et al. (2020) and Ammar et al. (2009) tested crude extracts of various DKE.

#### 4.4.2 DKE’s antibacterial activity

Among the included studies, antibacterial activity of 28 date palm cultivars was evaluated from 11 records, while two studies did not report cultivar information (Table 5). Among these, Ajwa, Khalas cultivars (3 studies each) and Deglet Nour (2 studies) were the most tested. The extraction process was well explained but varied among the studies. Water, ethanol, methanol, acetone, hexane and mixtures from these were the common solvents used for polyphenolic extractions (Table 4). Given that the choice of solvent is based on the nature of bioactive compound to be extracted, variations in the bioactive constituents based on the extraction protocols, solvent used, proportion of solvents in extraction mixture, solid: solvent ratio, extraction time, extraction temperatures, etc can be anticipated which may impact the antibacterial activity of DKE (Chassagne et al., 2021). Significant variations in bioactive content of extracts have been identified as an issue with standardization of phyto-pharmaceuticals and dietary supplements (Cirak and Radusiene, 2019). Similar problems were encountered in this systematic review and stratification of the antibacterial activity of DKE based on extraction method was not possible due to heterogeneity of protocols used in various studies. Similar challenges are reported while conducting meta-analysis of phytochemicals from oat and buckwheat (Raguindin et al., 2021).

**TABLE 5:** Overview of selected records reporting antibacterial activity of polyphenols sourced from date kernel extracts.

References	Cultivar (country)	Source information
Abuelgassim et al. (2020)	Khalas, Sukkari (Saudi Arabia)	Procured from local market in Riyadh
Ado et al. (2017)	ND (Nigeria)	Procured from Central Market, Kaduna, Voucher specimen (040,616) deposited at Dept of Applied Science herbarium, Kaduna Polytechnic, Kaduna
Al-Daihan and Bhat, (2012)	Mosaifah (Saudi Arabia)	plant collected from Riyadh
Ammar et al. (2009)	Belah Meghal (Egypt)	Obtained from El-Dakhla Oases, Voucher specimen (V-20) deposited at Pharmacognosy herbarium NRC, Egypt
Barakat et al. (2020)	Abreme (Egypt)	Obtained from Horticulture Institute Research, Agriculture Research Centre (ARC), Giza, Egypt. Voucher specimens (424–2018#) deposited in Botany department herbarium
Hussain et al. (2019)	Khalas, Abu Maan, Ajwa, Fard, Lulu, Mabroom, Khodari (UAE)	Purchased -Al Foah company, Al Saad, Al Ain, Abu Dhabi, UAE. Cultivar and origin Information based on the supplier reported information
Idris et al. (2017)	ND (Nigeria)	Procured from Central Market, Kaduna, Voucher specimen (040,616) deposited at Dept of Applied Science herbarium, Kaduna Polytechnic, Kaduna
Metoui et al. (2019)	Lemsi, Bouhattam, Amari, Hammouri, Korkobi, Matata, Halwaya, Rochdi, Deglet Nour, Baht, Eguiwa, Khadhouri (Tunisia)	Deglet Nour procured from the oasis of Kébéli while other cultivars collected at the “tamr stage” from coastal oasis of Gabes
Ouahioune et al. (2020)	Degla-Baida (Algeria)	Recovered as industrial waste from company Mehiri Dattes (Tolga city, Algeria: Latitude: 34°43′0″ N, Longitude: 5°22′0″ E, Altitude: 147 m)
Qasim et al. (2020)	Khalas, Ajwa (Tunisia)	Obtained from the local market of Faisalabad. Identified, classified, and approved from Botany Department, University of Agriculture, Faisalabad
Radfar et al. (2019)	Kabkab, Rabbi, Zahedi, Mazafati (Iran)	Provided by the Iranian Date Palm and Tropical Fruits Research Center, Ahwaz, Iran
Smaoui et al. (2020)	Deglet Nour (Tunisia)	Deglet Nour were collected from Kébéli (N: 33.4218°, E: 8.45754°) oasis on 2017 crop season
Selim et al. (2022)	Ajwa (Saudi Arabia)	Fruits in the “Tamar”stage collected from farm in Saudi Arabia’s Madinah region

The antibacterial activity of crude DKE was tested in 12 of the included 13 studies, however, Selim et al. (2022) tested for the Gallic Acid fraction of methanolic Ajwa-DKE. Hence, the results from this study were segregated and not included during data collation and was analysed separately.

Although the included studies tested antibacterial activity against several Gram-positive and Gram-negative bacteria (Figure 3B), for the purpose of comprehensive statistical analysis, data collation was performed for only those bacteria wherein DKE’s antibacterial activity was assessed against them in at least 3 of the selected records. Only 4 bacteria namely, *Escherichia coli*, *Staphylococcus aureus*, *Pseudomonas aeruginosa* and *Bacillus subtilis* met this criterion. Data for these organisms was collated to determine the median, 95% confidence intervals (CI) of mean, maximum, minimum, mean, standard deviations, etc of reported values.

#### 4.4.3 Effect on Gram-positive bacteria

Antibacterial activity for crude extracts of plants, if the ZOI is less than 12 mm is considered inactive, moderately active if ZOI is between 12 and 15 mm and assessed as active if ZOI is between 15 and 21 mm. A highly active crude extract has ZOI more than 18 mm (Saraiva et al., 2011; Silva et al., 2013; Voukeng et al., 2016; Le et al., 2020; Puvača et al., 2021).

**FIGURE 4:**
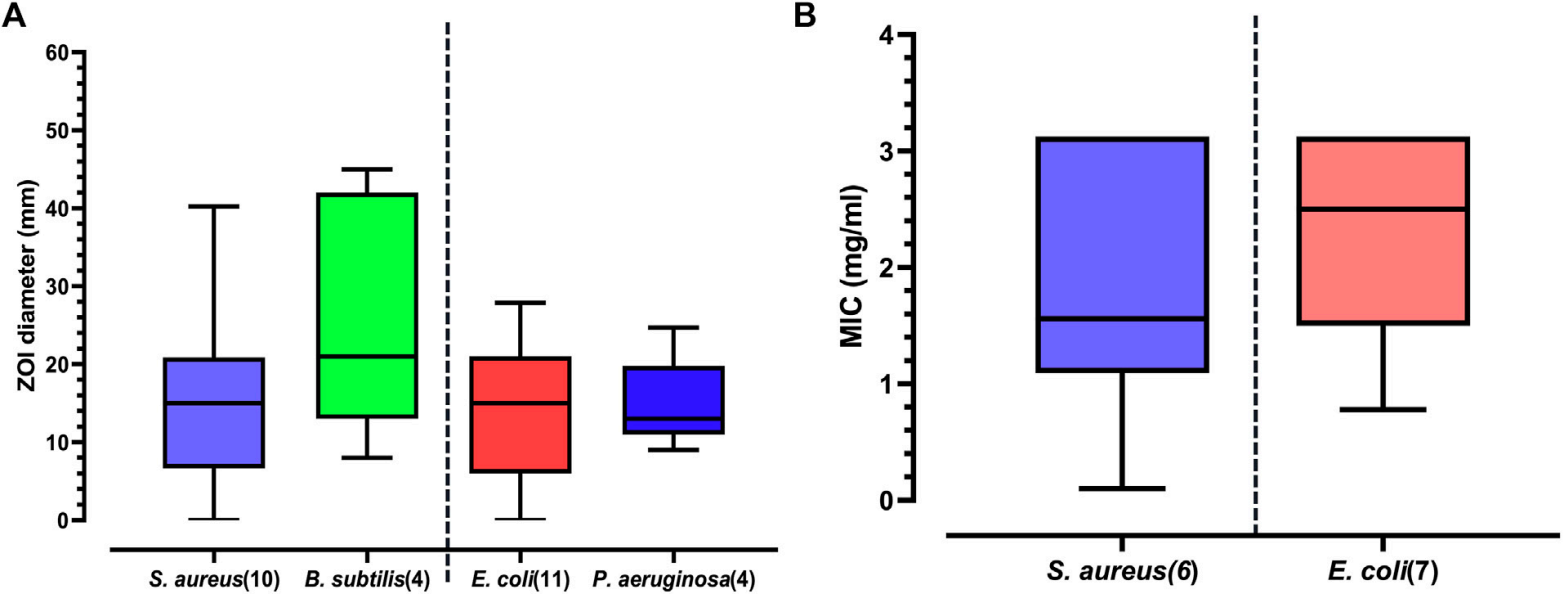
**(A)** Boxplot of pooled median ± range of zone of inhibition (ZOI) results reported in included studies against four pathogens gram positive: *S. aureus*, *B. subtilis* and gram negative: *E. Coli*, *P. aeruginosa* (values in parentheses indicate number of studies). **(B)** Boxplot of pooled median ± range of minimum inhibitory concentration (MIC) results reported against *S. aureus* and *E. Coli* in included studies (values in parentheses indicate number of studies).

The maximum reported ZOI against *S. aureus* was 40.25 mm (Smaoui et al., 2020) with 50% ethanolic extract of Deglet Nour cultivar while hexane or 50% ethanolic extract of Khalas and Khodari cultivars had no effect (Hussain et al., 2019). The median ZOI for *S. aureus* was estimated to be 15 mm (n = 86; Mean = 15.37 mm; Lower- Upper 95% CI of mean = 13.40–17.34 mm) matching the ZOI of 50% acetone extract and 1:1 Methanol: chloroform extracts of Abu Maan cultivar (Hussain et al., 2019) (Figure 4A; Supplementary Figure S2). Gallic acid is a common plant polyphenol which exhibits antimicrobial activity when tested alone or in combination with other natural products (Vandal et al., 2015; García-Pérez et al., 2019). Selim et al. (2022) reported the ZOI of Ajwa-DKE’s gallic acid fraction against *S. aureus* was 22 mm (Supplementary Figure S2) and this fraction had a greater inhibitory effect on Gram-positive bacteria than Gram-negative bacteria.

Antibacterial activity of DKE against *B. subtilis* assessed in four of the included studies (Figure 4A) when collated gave the maximum reported ZOI as 45 mm (Qasim et al., 2020) with 20% ethanolic extract of Ajwa cultivar; minimum was 8 mm (Ammar et al., 2009) with 70% methanolic extract of Belah Meghal cultivar and the median was 21 mm with 10% methanolic extract of Sukkari cultivar (Abuelgassim et al., 2020) (n = 7; Mean = 24.40 mm; Lower- Upper 95% CI of mean = 11.47–37.32 mm).

DKE’s antibacterial activity was also tested against some of the other Gram-positive bacteria. The ZOI values reported for *Enterococcus faecalis* were 21 ± 0.87 for Khalas cultivar and 20 ± 0.61 mm for Sukkari cultivar (Abuelgassim et al., 2020) while no activity was detected against this bacterium for any of the cultivars tested by Metoui et al. (2019). The ZOI values of Mosaifah-DKE reported for *Streptococcus pyrogenes* by Al-Daihan and Bhat (2012) were very low and could be considered as inactive as per classification for antibacterial activity. Selim et al. (2022) reported ZOI of 13 mm when gallic acid fraction of Ajwa-DKE was tested against *Streptococcus salivarius* indicating moderate activity of the DKE fraction. Smaoui et al. (2020) employed various solvent mixtures to identify an acetone: ethanol mix (1:1) which had the most antibacterial activity (ZOI 28.25 mm) against *Listeria monocytogenes.* Against *Staphylococcus epidermis*, the methanolic extract of Bouhattam DKE was reported to have the highest ZOI (21.33 mm) (Metoui et al., 2019).

#### 4.4.4 Effect on gram-negative bacteria

*E. coli* was the most employed Gram-negative test organism among the included studies. The aqueous extract of Korkobi cultivar was reported with the maximum ZOI of 27.87 mm (Metoui et al., 2019) while the solvent extracts of Khalas, Khodari, Kabkab, Rabbi, Zahedi and Mzafati cultivars did not exhibit any antibacterial activity against *E. coli* (Hussain et al., 2019; Radfar et al., 2019). The median ZOI for *E. coli* was estimated to be 15 mm (*n* = 86; Mean = 14.23 mm; Lower- Upper 95% CI of mean = 12.61–15.85 mm). This matched (Figure 4A, Supplementary Figure S3) the ZOI of 1:1 methanol: chloroform extract of Abu Maan cultivar and 50% acetone extract of Ajwa (Hussain et al., 2019). The ZOI for gallic acid fraction of Ajwa-DKE against *E. coli* was 11 mm Antibacterial activity against *P. aeruginosa*, tested in four studies gave a ZOI of 24.7 mm, 9 mm with aqueous extracts of Degla-Baida and Mosaifah cultivars respectively (Al-Daihan and Bhat, 2012). The median ZOI (Figure 4A; Supplementary Figure S3) for *P. aeruginosa* was 13 mm equivalent to ZOI of 10% methanolic extract of Sukkari cultivar (*n* = 7; Mean = 15.28 mm; Lower- Upper 95% CI of mean = 10.03–20.54 mm (Abuelgassim et al., 2020). The gallic acid fraction of Ajwa-DKE showed no antibacterial activity against *P. aeruginosa* (Selim et al., 2022).

Similarly for Gram-negative organisms such as *P. fluorescens* the antibacterial activity of Belah Meghal-DKE was reported (Ammar et al., 2009) to be very low (ZOI 7 ± 0.23 mm) which can be considered as inactive. In case of *S. enterica* the highest ZOI value was reported for Deglet Nour-DKE of 46 mm using a mixture of solvents (Smaoui et al., 2020). The results of ZOI against *S. typhimurium* showed inactive level for aqueous extracts, moderate to high activity for non-polar solvent-based extracts (Metoui et al., 2019) obtained from various cultivars of DKE. The high activity against *S. typhimurium* using methanolic extracts was corroborated for Khalas-DKE and Sukkari-DKE by Abuelgassim et al. (2020). Similarly, Ouahioune et al. (2020) reported a ZOI of 18 mm for Degla Baida-DKE against *K. pneumonia*. Qasim et al. (2020) reported ZOI of 33 ± 3.1 mm (Ajwa-DKE) and 31 ± 2.8 mm (Khalas-DKE) when tested against *P. multocida* which indicates highly active antibacterial action of the extracts.

The reported ZOI values indicate the antibacterial activity of polyphenolic DKE ranges from inactive (ZOI <12 mm) for several DKE. Those DKE which showed effect generally ranged from moderately active (ZOI 12–15 mm) and highly active (ZOI >18 mm) subject to the extraction method and cultivars tested (Puvača et al., 2021). The higher antibacterial activity against Gram-positive microorganisms was reported typically for solvent (less polar) based DKE. The aqueous DKE seem to provide moderate to high antibacterial activity against Gram-negative microorganisms. Thus, the antibacterial activity is largely governed by the test microorganism employed (Ammar et al., 2009; Al-Daihan and Bhat, 2012; Metoui et al., 2019; Abuelgassim et al., 2020; Ouahioune et al., 2020; Qasim et al., 2020; Smaoui et al., 2020; Selim et al., 2022). Furthermore, the reported antibacterial activity of DKE against some of the ESKAPE pathogens are promising and warrants further investigation. Altogether, these findings highlight the potential to extract polyphenolic compounds using different solvents/solvent-mixtures, that have antibacterial activity against specific microorganisms.

#### 4.4.5 Minimum inhibitory concentration (MIC)

The diverse test conditions (Table 3) employed in the included studies challenged effective inter-laboratory data comparison. The choice of the medium, pH, agar depth, incubation conditions, inoculum density, etc impact the MIC values (Bubonja-Šonje et al., 2020). Hence, MIC values could be collated for only *S. aureus* and *E. Coli,* which met our pre-set criterion. The median, maximum, minimum reported values and the 95% confidence intervals (CI) of mean were calculated. Crude plant extracts can be classed as highly active if MIC <0.1 mg/ml; active if between 0.1 and0.5 mg/ml; moderately active if between 0.5 and1.0 mg/ml; weak if between 1.0 and8.0 mg/ml and inactive if MIC >8.0 mg/ml against those bacteria (Puvača et al., 2021).

**FIGURE 5:**
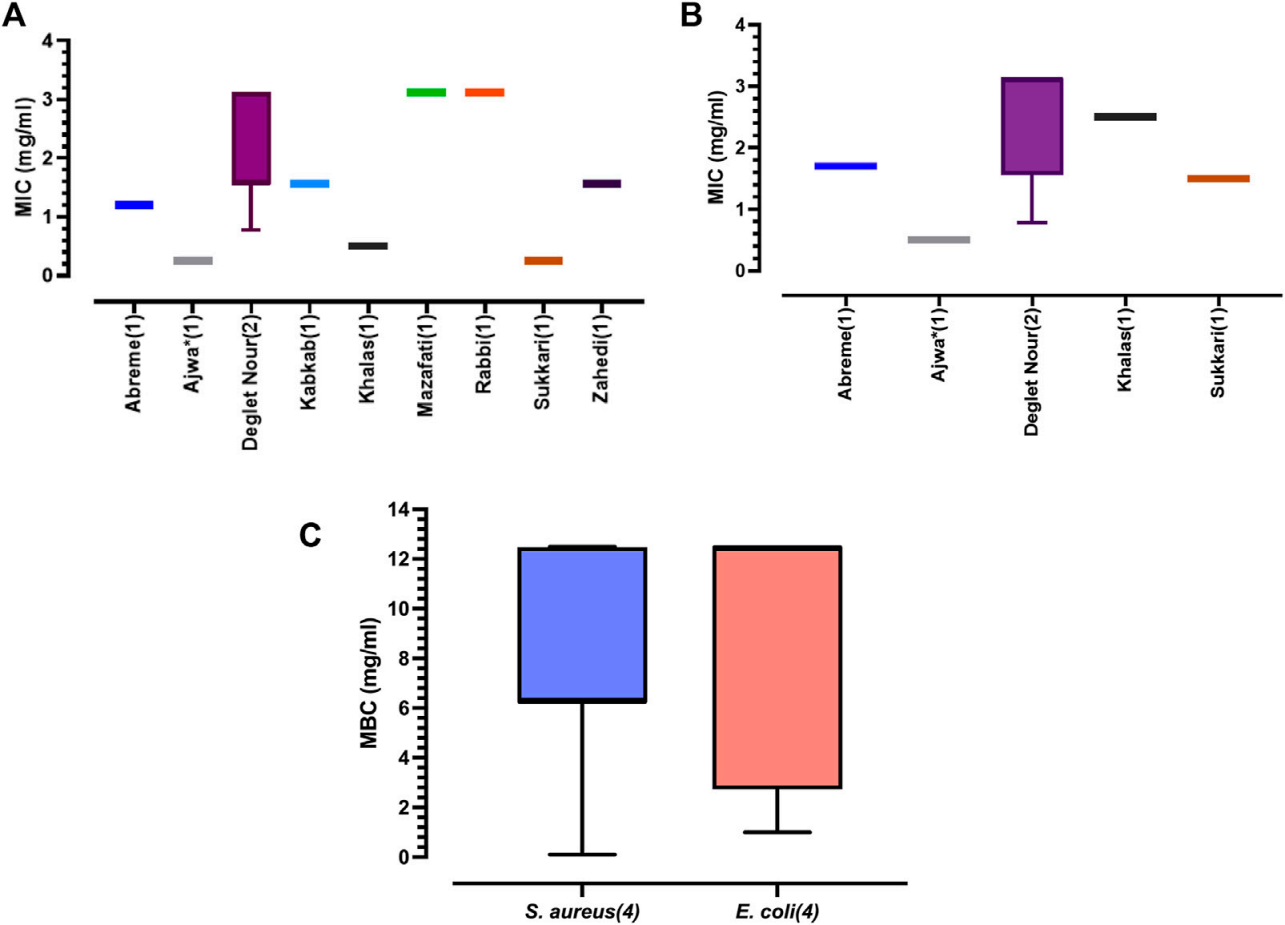
**(A)** Boxplot of pooled median ± range of minimum inhibitory concentration (MIC) results reported against *S. aureus* in included studies (values in parentheses indicate number of studies). *Gallic acid was isolated from the date seed extract and used for the test. **(B)** Boxplot of pooled median ± range of minimum inhibitory concentration (MIC) results reported *E. coli* in included studies (values in parentheses indicate number of studies). *Gallic acid was isolated from the date seed extract and used for the test. **(C)** Boxplot of pooled median ± range of minimum bactericidal concentration (MBC) results reported against *S. aureus* and *E. coli* in included studies (values in parentheses indicate number of studies).

The MIC for *S. aureus* ranged from 0.1 to 3.13 mg/ml (Idris et al., 2017; Smaoui et al., 2020), considered weakly active to active (Puvača et al., 2021) when ethanolic extract of unknown cultivar and Deglet Nour-DKE, respectively, were applied. The median MIC for *S. aureus* was 1.56 mg/ml for Kabkab cultivar’s ethanolic extract (Radfar et al., 2019) (n = 18; Mean = 1.72 mg/ml; Lower- Upper 95% CI of mean = 1.21–2.22 mg/ml). Selim et al. (2022) reported the MIC of gallic acid fraction of Ajwa-DKE against *S. aureus* to be 0.25 mg/ml (Figure 4B; Figure 5A).

The reported MIC against *E. Coli* ranged from 0.78 to 3.13 mg/ml for Deglet Nour-DKE (Smaoui et al., 2020), considered to be weakly active to moderately active (Puvača et al., 2021) with a calculated median MIC of 2.5 mg/ml (*n* = 15; Mean = 2.23 mg/ml; Lower- Upper 95% CI of mean = 1.71–2.76 mg/ml) which matched the reported MIC for Khalas-DKE (Abuelgassim et al., 2020). The lowest MIC was 0.78 mg/ml (Smaoui et al., 2020) for 4.7: 1: one acetone: ethanol: water extract of Deglet Nour cultivar. Selim et al. (2022) reported the MIC for gallic acid fraction of Ajwa-DKE against *E. coli* to be 0.5 mg/ml (Figure 4B, Figure 5B).

In case of pure compounds, Hossan et al. (2018) suggest classification as highly active (MIC <0.01 mg/ml), moderately active (MIC = 0.01–0.1 mg/ml) and low activity (MIC >0.1 mg/ml). The gallic acid fraction of Ajwa-DKE (pure compound) when tested against *S. aureus* and *E. coli* showed MIC values of 0.25 mg/ml and 0.5 mg/ml equating to low activity (Figures 5A, B). Therefore, further studies are required where pure compounds separated from crude DKE are tested similar to Selim et al. (2022) for their antibacterial activity. The MIC value of the collated data also indicates that crude-DKE’s are more active against Gram-positive bacteria such as *S. aureus* than against the Gram-negative bacteria such as *E. coli* (Bouarab-Chibane et al., 2019; Zhang et al., 2021).

#### 4.4.6 MBC and MBC/MIC ratio

MBC establishes the lowest concentration of DKE required to kill a particular bacterium (Górniak et al., 2019). MBC results were collated where a minimum of three studies are available for the test organism. This criterion limited MBC data collation to only *S. aureus* and *E. Coli* and pooled data was used to determine median, 95% CI of mean, mean, maximum and minimum values (Figure 5C). MBC for *S. aureus* was highest at 12.5 mg/ml (Radfar et al., 2019) with 80% ethanolic extracts of Mazafati and Rabbi cultivars, while lowest at 0.1 mg/ml (Idris et al., 2017) with unknown cultivar (sourced from Kaduna State, Nigeria). Among the cultivars tested in the included studies, mixed solvent Deglet Nour-DKE (with higher proportion of acetone) had the lowest MBC against *S. aureus* of 1.56 mg/ml (Smaoui et al., 2020). The median MBC for *S. aureus* was 6.25 mg/ml (*n* = 15; Mean = 7.4 mg/ml; Lower- Upper 95% CI of mean = 5.09–9.71 mg/ml) similar to that reported by Radfar et al. (2019) using ethanolic extract of Zahedi cultivar. Deglet Nour-DKE extracted using a variety of solvents such as acetone, ethanol and/or their mixtures were all evaluated to have MBC values of 6.25 mg/ml by Smaoui et al. (2020). The MBC for gallic acid fraction of Ajwa-DKE (Selim et al., 2022) tested against *S. aureus*, was 0.25 mg/ml (Figure 5C), data not shown separately.

The highest reported MBC value for *E. coli* was reported by (Smaoui et al., 2020) to be 12.48 mg/ml from Deglet Nour cultivar while the lowest reported was 1.56 mg/ml for Deglet Nour-DKE extract prepared using 4.7:1:1 (acetone: ethanol: water) mixed solvent (Smaoui et al., 2020) similar to other reports (Ado et al., 2017; Idris et al., 2017). The median values calculated from reported data was 12.48 mg/ml (*n* = 12; Mean = 8.62 mg/ml; Lower- Upper 95% CI of mean = 5.40–11.83 mg/ml). The MBC for gallic acid fraction of Ajwa-DKE (Selim et al., 2022) tested against *E. coli*, was reported to be 0.5 mg/ml (Figure 5C), data not separately shown.

The effect of an antibacterial agent is considered as bactericidal if the MBC/MIC ratio is ≤ 4, otherwise as bacteriostatic (Thomas et al., 2012). The MBC/MIC ratios could only be calculated for *S. aureus* and for *E. coli* as there were four included studies for each organism and the ratios for both were between 1 and 4, implying a potential bactericidal effect of crude DKE against these two pathogens. Further research into the antibacterial activity of polyphenolic compounds from DKE can aid in valorising and bioprospecting compounds from date kernel.

#### 4.4.7 Stratification of DKE cultivars based on their antibacterial activity

Stratification of DKE’s antibacterial activity according to cultivars was set as one of the objectives. Cultivar associated variations in reported ZOI values against *S. aureus* and *E. coli* (Supplementary Figures S2, S3), respectively. Abuelgassim et al. (2020) reported that *E. coli* was resistant to Sukkari- DKE (no impact reported) but was sensitive to Khalas- DKE for the dosages tested. DKE’s extracted from Kabkab, Khodari, Mazafati, Rabbi and Zahedi cultivars were also reported to be ineffective in inhibiting *E. coli* (Hussain et al., 2019; Radfar et al., 2019). However, based on the reported ZOI (Supplementary Figure S3), for DKE’s from Hammouri, Korkobi, Baht and Bouhattam cultivars, they showed potential antibacterial activity (Metoui et al., 2019). DKE’s from Sukkari and Khalas cultivars were reported to be more effective than DKE’s from Mazafati and Rabbi cultivars (Figure 5A) against *S. aureus*. The gallic acid fraction of Ajwa-DKE showed greater inhibition activity against *E. coli* compared to the crude DKE’s from other cultivars (Figure 5B).

Above findings suggest variability in antibacterial activity depending upon the source cultivar for DKE. Similar variability has been reported in antibacterial properties of date palm fruits (Samad et al., 2016), burbark cactus extracts (Mabotja et al., 2021) and mint extracts (Kowalczyk et al., 2021). Cultivar associated differences in polyphenolic content and profile of DKE has been reported in literature (Bouhlali et al., 2020) which may contribute to the variability in the antibacterial activity observed. Additional studies to bio prospect fractions of polyphenolic compounds from DKE for their pathogen specific antibacterial activity is required considering the variety of date palm cultivars available.

#### 4.4.8 Limitations

This systematic review was challenging due to variations in the test methodology, heterogeneity of data, and incomplete information. We anticipate limitations from exclusion of studies not fulfilling the eligibility criteria, limitations arising from inclusion of studies with medium Risk of Bias, academic databases excluded from our search, Boolean keywords and combinations chosen, defined aims of study, and inclusion/exclusion criteria applied. Finally, the authors were limited due to language bias, and selected only English reports published until March 2022. Any relevant publications in other languages were not included in this review.

## 5 Conclusion

The body of available literature suggests that DKE can be a potential source of antibacterial polyphenolic compounds. This systematic review evaluated 888 publications that met the search criteria and based on the eligibility criteria, thirteen records were further analysed for polyphenolic and antibacterial measurements. A total of 28 date palm cultivars was evaluated for their antibacterial activity among which Ajwa and Khalas cultivars were the most studied. Qualitative and quantitative phytochemical analysis established the presence of polyphenolic compounds in DKE. The total polyphenolic content varied from 0.05 to 229.7 mg GAE g^−1^ DW among the included studies. The range of measured polyphenol content of DKE is attributed to differences in date palm cultivars, extraction methods including solvents and sample-solvent ratios, *etc.* The polyphenols from DKE varied in their antibacterial activity and tended to be moderately active to highly active against Gram-positive microorganisms while showing weakly active to moderately active effect against Gram-negative bacteria. The low polar solvent (ethanol, methanol, etc) based extracts of Deglet Nour and Ajwa cultivars were highly active against Gram-positive bacteria namely *S. aureus* and *B. subtilis* while the aqueous extracts of date kernels from Korkobi and Degla-Baida cultivars were highly active against Gram negative bacteria such as *E. coli* and *P. aeruginosa*. Thus, natural polyphenols can be sourced from date kernels that have potential antibacterial activity. However further studies on purified fractions isolated from crude DKE and their *in-vitro* and *in-vivo* therapeutic applicability on ESKAPE pathogens is required. This study highlights DKE as an excellent bioprospecting source for antibacterial compounds.

## Data Availability

The original contributions presented in the study are included in the article/Supplementary Material, further inquiries can be directed to the corresponding author.

## Supplementary material

SUPPLEMENTARY FIGURE S1 Boxplot of pooled median ± range of total phenolic content (TPC) values reported in included studies (values in parentheses indicate number of studies by cultivar).

SUPPLEMENTARY FIGURE S2 Boxplot of pooled median ± range of zone of inhibition (ZOI) results reported against S. aureus in included studies (values in parentheses indicate number of studies). *Gallic acid was isolated from the date seed extract and used for the test.

SUPPLEMENTARY FIGURE S3 Boxplot of pooled median ± range of zone of inhibition (ZOI) results reported against E. coli in included studies (values in parentheses indicate number of studies). *Gallic acid was isolated from the date seed extract and used for the test.
