# Supplementary figures and images for: A systematic review of antibacterial activity of polyphenolic extract from date palm (Phoenix dactylifera L.) kernel
Source: Front Pharmacol. 2023 Jan 10;13:1043548. doi: 10.3389/fphar.2022.1043548 (PMC9871312; doi:10.3389/fphar.2022.1043548)

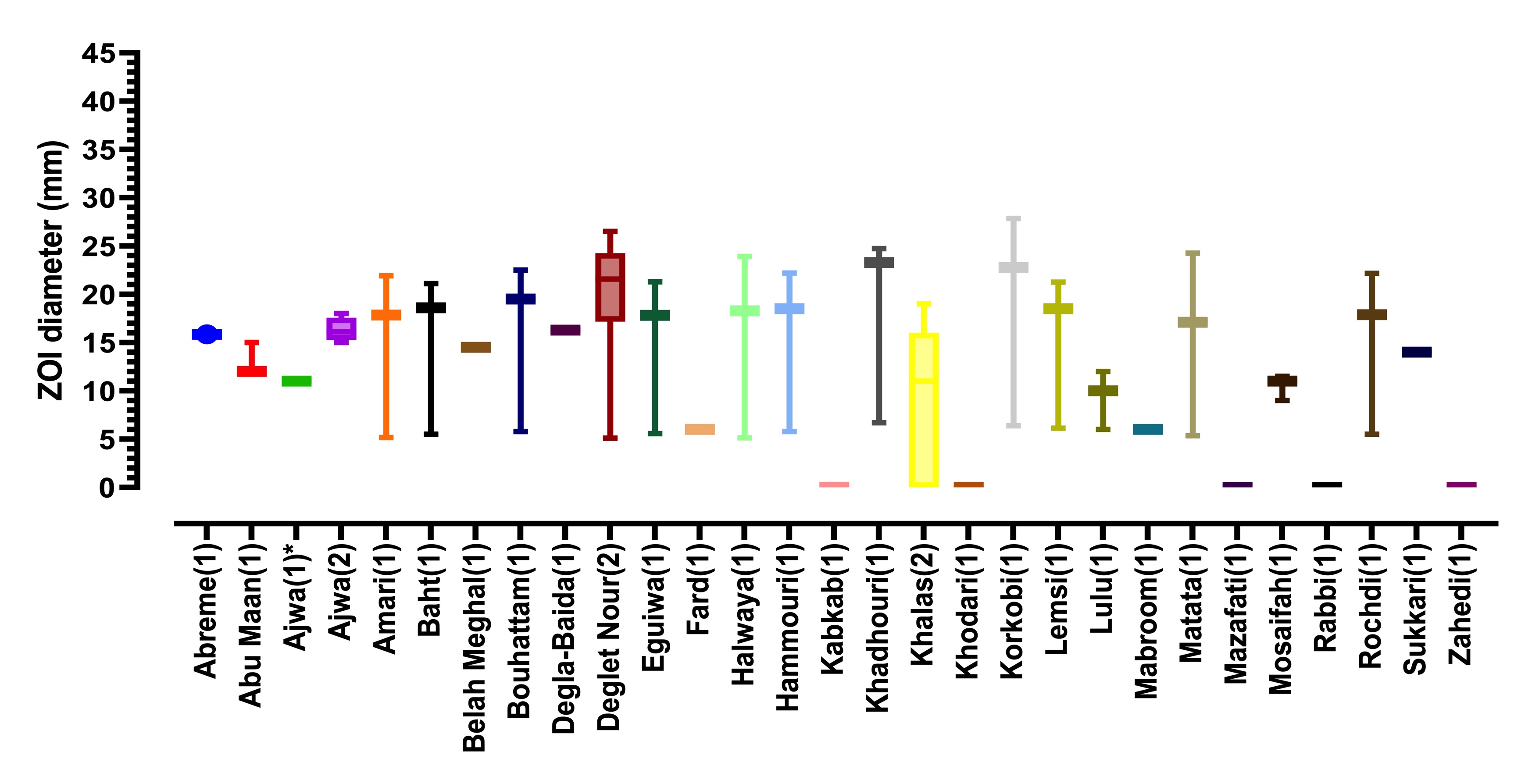

Supplement: Supplementary file 1 [file Image3.JPEG]

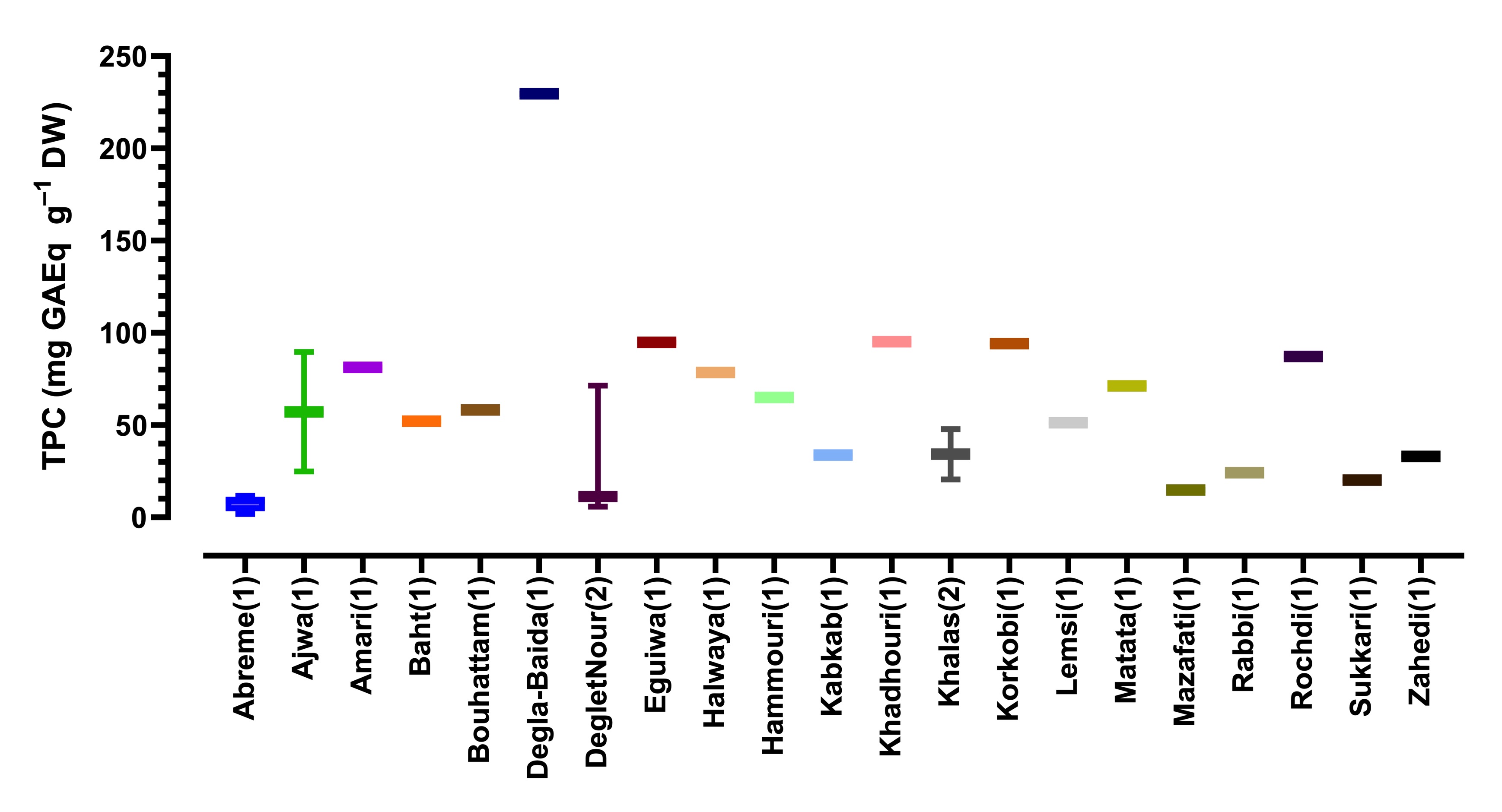

Supplement: Supplementary file 2 [file Image1.JPEG]

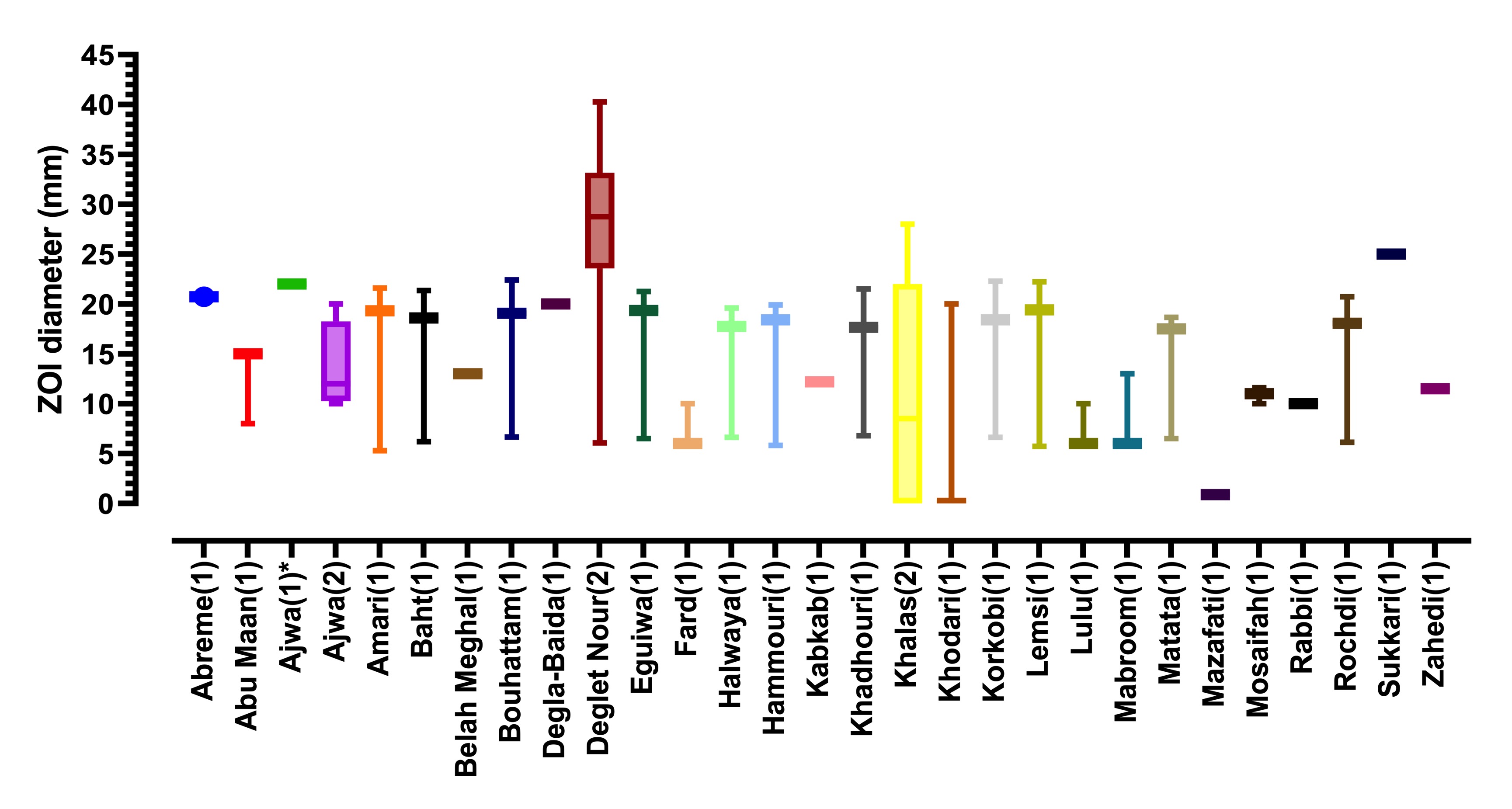

Supplement: Supplementary file 3 [file Image2.JPEG]
